# Risk Assessment Models for Predicting Venous Thromboembolism in Patients with Pancreatic Cancer

**DOI:** 10.3390/cancers17040597

**Published:** 2025-02-10

**Authors:** Corinne Frere, Sophie Gourgou, Audrey Winter, Ludovic Gauthier, Cindy Canivet, Benjamin Crichi, Zora Marjanovic, Alexandra Yannoutsos, Okba Bensaoula, Louis Buscail, Barbara Bournet, Dominique Farge

**Affiliations:** 1 Department of Hematology, Pitié-Salpêtrière Hospital, Assistance Publique-Hôpitaux de Paris, F-75013 Paris, France; 2 Sorbonne Université, Faculty of Medicine, INSERM UMRS 1166, GRC 27 GRECO, F-75013 Paris, France; 3 Biometrics Unit, Montpellier Cancer Institute, University of Montpellier, F-34090 Montpellier, France; sophie.gourgou@icm.unicancer.fr (S.G.); audrey.winter@icm.unicancer.fr (A.W.); ludovic.gauthier@icm.unicancer.fr (L.G.); 4 Department of Gastroenterology, Toulouse University Hospital, F-31400 Toulouse, France; canivet.c@chu-toulouse.fr (C.C.); buscail.l@chu-toulouse.fr (L.B.); bournet.b@chu-toulouse.fr (B.B.); 5 Internal Medicine Unit (04): CRMR MATHEC, Maladies Auto-Immunes et Thérapie Cellulaire, Centre de Référence des Maladies Auto-Immunes Systémiques Rares d’Ile-de-France, Saint-Louis Hospital, Assistance Publique-Hôpitaux de Paris, F-75010 Paris, France; benjamin.crichi@aphp.fr (B.C.); dominique.farge-bancel@aphp.fr (D.F.); 6 Department of Hematology, Saint-Antoine Hospital, Assistance Publique-Hôpitaux de Paris, F-75012 Paris, France; zora.marjanovic@aphp.fr; 7 Department of Vascular Medicine, Hôpital Paris Saint-Joseph, F-75014 Paris, France; ayannoutsos@ghpsj.fr; 8 Institut Curie, F-92210 Saint-Cloud, France; okbaibn-nafaa.bensaoula@curie.fr; 9 Toulouse University, The Toulouse Cancer Research Center, INSERM UMRS 1037, F-31100 Toulouse, France; 10 Université Paris Cité, Faculty of Medicine, IRSL, Recherche Clinique en Hématologie, Immunologie et Transplantation, URP3518, F-75010 Paris, France

**Keywords:** venous thromboembolism, pancreatic cancer, risk assessment model

## Abstract

Background: Data on the performance of the Khorana, PROTECHT, and ONKOTEV risk assessment models (RAMs) to predict venous thromboembolism (VTE) in patients with pancreatic cancer (PC) receiving outpatient chemotherapy remain limited. We performed a head-to-head comparison of these RAMs in patients with newly diagnosed PC enrolled in the nationwide, multicenter, and prospective BACAP cohort. Methods: The Khorana, PROTECHT, and ONKOTEV scores were calculated at enrollment prior to chemotherapy. Patients were stratified into intermediate- and high-VTE-risk groups according to each RAM. The primary study outcome was VTE at a 6-month follow-up. The accuracy and discriminatory performance of the scores were assessed by calculating time-dependent Brier scores and c-indexes. Sub-distribution hazard ratios (SHRs) between high- and intermediate-risk patients were estimated. Results: Of 762 PC patients, 73 developed VTE within 6 months. In the competing risk analysis, the cumulative incidence of VTE at 6 months was 16.4% (95% CI, 13.8–19.1). The time-dependent Brier score was 0.14 (95% CI, 0.12–0.15) for all scores, indicating well-calibrated predictions. The respective time-dependent c-index of the Khorana, the PROTECHT, and the ONKOTEV scores was 0.50 (95% CI, 0.46–0.55), 0.50 (95% CI, 0.49–0.51), and 0.53 (95% CI, 0.48–0.58), indicating poor discrimination. The SHRs between high- and intermediate-risk patients ranged from 1.05 (95% CI, 0.76–1.44) for the ONKOTEV score to 1.06 (95% CI, 0.77–1.45) for the Khorana score. Conclusion: In newly diagnosed PC patients receiving outpatient chemotherapy, the Khorana, PROTECHT, and ONKOTEV scores demonstrated a poor performance in predicting VTE at 6 months, highlighting the need for new tools to guide thromboprophylaxis decisions.

## Simple Summary

Data on the performance of the Khorana, PROTECHT, and ONKOTEV risk assessment models (RAMs) to predict venous thromboembolism (VTE) in patients with newly diagnosed pancreatic cancer (PC) receiving outpatient chemotherapy remain limited. The nationwide, multicenter, and prospective BACAP cohort study allowed for a head-to-head comparison of all three RAMs to predict VTE at 6 months in 762 newly diagnosed PC patients receiving ambulatory chemotherapy. In the competing risk analysis, the 6-month cumulative incidence of VTE was 16.4% (95% CI, 13.8–19.1). The respective time-dependent c-index of the Khorana, PROTECHT, and ONKOTEV scores were 0.50 (95% CI, 0.46–0.55), 0.50 (95% CI, 0.49–0.51), and 0.53 (95% CI, 0.48–0.58), indicating poor discrimination. In this head-to-head comparison, the Khorana, PROTECHT, and ONKOTEV scores demonstrated poor performance to predict VTE at 6 months in newly diagnosed PC patients receiving outpatient chemotherapy, highlighting the need for new tools to guide thromboprophylaxis decisions.

## 1. Introduction

Pancreatic ductal adenocarcinoma (PDAC), often referred to as pancreatic cancer (PC), is a highly aggressive malignancy with a 5-year survival rate lower than 13% [1]. Its incidence is increasing worldwide, and PC is projected to become the second leading cause of cancer-related mortality in the United States by 2026 [2]. Approximately 80% of newly diagnosed PC patients have locally advanced or metastatic disease that precludes curative surgery. In most cases, chemotherapy and supportive care are the only therapeutic options to improve survival and quality of life.

Venous thromboembolism (VTE) is a common but preventable cause of morbidity and mortality in cancer patients, and PC is associated with the highest risk of VTE of any malignancy [3]. Importantly, patients with early-onset VTE after PC diagnosis have significantly decreased progression-free and overall survivals compared to those without VTE, as we and others have previously reported [4,5,6,7,8,9,10]. Pharmacological thromboprophylaxis reduces the risk of VTE [11], but is still not widely used in this cancer patient population due to concerns about bleeding risk and lack of demonstrated survival benefit.

In this context, several risk assessment models (RAMs) have been developed to identify cancer patients who are at higher risk for VTE when undergoing ambulatory chemotherapy. The most widely used and best validated RAM is the Khorana score, which comprises five items: site of cancer, pre-chemotherapy platelet count ≥ 350 × 10^9^/L, pre-chemotherapy hemoglobin level <10 g/dL or use of erythropoiesis-stimulating agents, pre-chemotherapy leukocyte count >11 × 10^9^/L, and body mass index ≥ 35 kg/m^2^ [12]. Cancer patients with 0 points are classified as low risk, 1 to 2 points as intermediate risk, and ≥3 points as high risk for VTE.

Given that the Khorana score assigns 2 points for PC, it classifies all PC patients as having at least an intermediate risk of VTE, and whether its use is relevant in PC patients appears questionable. Other RAMs, such as the PROTECHT [13] and ONKOTEV [14] scores, incorporate additional relevant items, notably the use of cytotoxic chemotherapy or cancer stage. Data on the performance of these RAMs in PC patients remain limited [15], and no prospective head-to-head comparison of these RAMs has been performed yet.

The aim of the present study was to compare the accuracy and discriminatory performance of the Khorana, the PROTECHT, and the ONKOTEV scores to predict the risk of VTE at 6 months in patients with newly diagnosed PC of any stage receiving outpatient chemotherapy.

## 2. Materials and Methods

### 2.1. Study Design and Participants

The nationwide, multicenter, and prospective Base Clinico-Biologique de l’Adénocarcinome Pancréatique (BACAP) cohort study (ClinicalTrials.gov Identifier NCT02818829) is an ongoing collaborative project involving 15 French oncology centers [8,16]. Briefly, consecutive adult patients with histologically and/or cytologically proven PDAC of any stage were prospectively enrolled at PC diagnosis prior to any treatment initiation. Participants were managed according to the ESMO clinical practice guidelines for the diagnosis and treatment of PC until last follow-up or death [17]. The detailed BACAP study design has been reported previously [8,16].

For the present analysis, all BACAP participants were eligible if they were scheduled to start outpatient chemotherapy within 30 days. Participants with VTE at the time of enrollment or within 3 months prior to enrollment were excluded. None of the patients included in this study received routine thromboprophylaxis. The inclusion period spanned from May 2014 to July 2020.

The BACAP project was approved by the relevant national ethics committee (CPP Sud-ouest et Outre-Mer I, March 2014). All patients provided written informed consent to participate. This report conforms to the Transparent Reporting of a Multivariable Prediction Model for Individual Prognosis or Diagnosis (TRIPOD) statement [18].

Demographic, clinical, laboratory, radiological, and clinical outcome data were collected at enrollment and at routine follow-up using the Ennov Clinical^®^ software version 8.2.310 (Paris, France), with regular data quality control [8,16].

The primary outcome was incident VTE, defined as a composite of objectively confirmed symptomatic or incidental pulmonary embolism (PE), symptomatic proximal or distal lower-extremity deep vein thrombosis (DVT), non-catheter-related symptomatic upper-extremity DVT, symptomatic catheter-related thrombosis, or symptomatic or incidental visceral venous thrombosis (VVT) including splenic/superior mesenteric/portal vein thrombosis. Patients were not routinely screened for VTE. The VTE diagnosis was initially established by the referring physician based on imaging results (compression ultrasound scanning, multidetector computed tomography, ventilation/perfusion lung scintigraphy, pulmonary angiography) and adjudicated by a committee of independent trained experts. The occurrence, timing, and type of VTE were obtained from the electronic database.

### 2.2. Risk Assessment Models

The Khorana [12], the PROTECHT [13], and the ONKOTEV [14] scores were calculated at enrollment prior to chemotherapy (Table S1 in Supplementary Material). According to the Khorana score categorization, patients with 2 points were classified as intermediate risk, and those with ≥3 points as high risk for VTE [12]. The PROTECHT score was calculated using the Khorana score items plus 1 additional point for gemcitabine-based therapy and platinum-based chemotherapy (or 2 points for the association), and then by summing all points [13]. According to the PROTECHT score categorization, patients with 2 points were classified as intermediate risk, and those with ≥3 points as high risk for VTE [13]. The ONKOTEV score was calculated by assigning 1 point for each of the following items: a Khorana score > 2, previous VTE, metastatic disease, and macroscopic vascular or lymphatic compression by the tumor, and then summing the points [14]. The ONKOTEV risk groups were dichotomized as follows: patients with <2 points were classified as intermediate risk and those with ≥2 points as high risk for VTE.

### 2.3. Statistical Analysis

Baseline patient characteristics were summarized using standard descriptive statistics. Each RAM was evaluated using a Fine and Gray regression model, with VTE as outcome and death as a competing risk [19]. Patients were censored if they underwent cancer surgery or at last follow-up or death.

A multivariate Cox proportional hazards model with a time-dependent covariate was developed to identify risk factors for VTE by using a stepwise selection process. Variables associated with a *p* value < 0.10 in univariate analysis.

Multiple imputation with predictive mean matching was used for sporadic missing values to calculate the VTE risk scores in the entire study population [20]. The imputation model included all baseline characteristics and outcome data to generate 30 imputed datasets. Model parameter estimates along with standard errors were pooled using Rubin’s rules to account for imputation variability.

The accuracy of the scores in predicting VTE at a 6-month follow-up was evaluated by computing a time-dependent version of the Brier score [21], consistent with censoring and event times, which incorporates both discrimination and calibration, with corresponding 95% confidence intervals (CIs) [22]. The discriminatory performance of each score was evaluated by computing the 6-month time-dependent c-index with its corresponding 95% CI.

The numbers needed to treat (NNT) with corresponding 95% CIs were estimated using a previously described formula [23]. For each risk category i: NNT = 1/[(1 − ${Cumulative\;incidence\;at\;6\;months}_{i}$)^RR^) − (1 − ${Cumulative\;incidence\;at\;6\;months}_{i}$)]. The RR used was reported in a recent pooled analysis [24] of CASSINI [25] and AVERT [26].

All statistical analyses were performed using Stata, version 16.0 (StataCorp LLC, College Station, TX, USA) and R, version 4.2.1 (R Project for Statistical Computing) using “cmprisk”, “SurvMetrics”, and “riskRegression” packages.

## 3. Results

### 3.1. Population Characteristics

**Figure 1:**
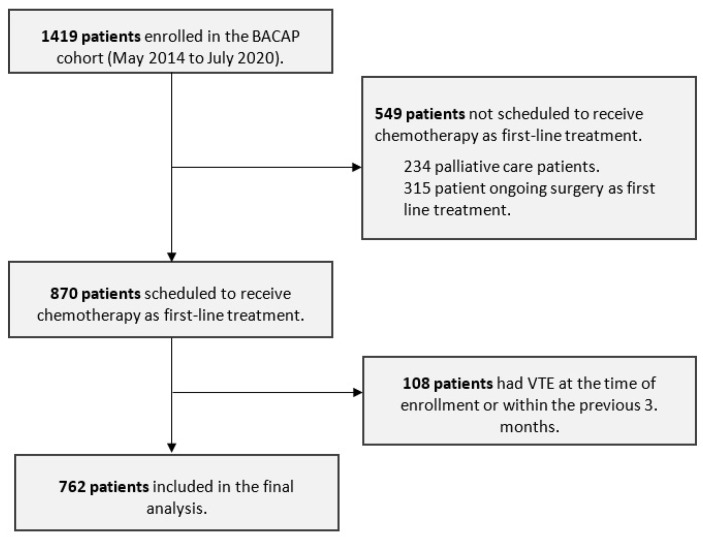
Flow chart of the study.

Between May 2014 and July 2020, 870 out of 1419 newly diagnosed PC patients were prospectively enrolled in the BACAP cohort and scheduled to receive outpatient chemotherapy as first-line treatment. After excluding 108 patients (12.4%) with VTE at the time of enrollment or within three months prior to enrollment, 762 patients were included in the analysis (Figure 1).

The baseline characteristics of the patients are described in Table 1.

Patient median age was 69 years (interquartile range, 60–76) and 412 (54.1%) were male. A total of 285 patients (37.6%) had metastatic disease and 317 (47.3%) had macroscopic vascular or lymphatic compression. Most patients received neoadjuvant therapy with platinum-based therapy (61.0%), gemcitabine-based therapy (33.9%), or both (3.8%) within 6 months.

### 3.2. Outcomes

During a median follow-up of 40 months (95% CI, 34.3–46.8), 195 (25.6%) patients developed one or more VTE events (Table S2 in Supplementary Material) and 354 (46.5%) died. The cumulative incidence of VTE and death is shown in Figure S1 (in Supplementary Material). Within the first 6 months after PC diagnosis, 73 patients developed VTE, 17 (23.3%) had PE, 17 (23.3%) DVT, 29 (31.5%) VVT, 4 (5.5%) catheter-related thrombosis, and 6 (8.2%) combined VTE events (Table 2), and 261 patients died. A total of 41 (56.2%) VTE events were diagnosed incidentally on routine imaging for treatment response evaluation or cancer restaging (Table 2). In the competing risk analysis, the 6-month cumulative incidence of VTE was 16.4% (95% CI, 13.8–19.1). Older age (≥70 years; HR, 0.63; 95% CI, 0.47–0.84) and metastatic disease (HR, 2.11; 95%CI, 1.22–3.63) were significantly associated with the risk of VTE in multivariate analyses (Table S3 in Supplementary Material).

Data to calculate the Khorana, the PROTECHT, and the ONKOTEV scores were available for 697 (91.5%), 697 (91.5%), and 618 (81.1%) patients, respectively. The distribution of the scores is shown in Table S4 (Supplementary Material). The dichotomized Khorana score classified 501 (65.8%) patients as intermediate risk (Khorana score 2) and 196 (25.7%) as high risk for VTE (Khorana score ≥ 3). The dichotomized PROTECHT score classified 7 (1.0%) patients as intermediate risk (PROTECHT score 2) and 690 (99.0%) as high risk for VTE (PROTECHT score ≥ 3). Finally, the dichotomized ONKOTEV score classified 407 (65.9%) patients as intermediate risk (ONKOTEV score < 2) and 211 (34.1%) as high risk for VTE (ONKOTEV score ≥ 2).

### 3.3. Accuracy and Discriminatory Performance of the Scores to Predict the 6-Month Risk of VTE

Results are summarized in Table 3 and Figure S2 (Supplementary Material). For the Khorana score, the time-dependent Brier score (overall accuracy) was 0.14 (95% CI, 0.12–0.15), meaning that predictions were well calibrated, while the time-dependent c-index was 0.50 (95% CI, 0.46–0.55), indicating poor discrimination. Using a positivity threshold of 3 points, the 6-month cumulative VTE incidence was 16.1% (95% CI, 11.4–21.5) in high-risk patients versus 16.5% (95% CI, 13.4–19.8) in intermediate-risk patients (SHR, 1.06; 95% CI, 0.77–1.45; *p* = ns).

For the PROTECHT score, the time-dependent Brier score was 0.14 (95% CI, 0.12–0.15), with good agreement between predicted and observed values, while the time-dependent c-index was 0.50 (95% CI, 0.49–0.51), indicating poor discrimination. Using a 3-point positivity threshold, high-risk patients had a 6-month cumulative VTE incidence of 16.5% (95% CI, 13.9–19.3), while intermediate-risk patients had no VTE events (SHR, 1.87; 95% CI, 0.29–12.05; *p* = ns).

Using either the Khorana or PROTECH scores, the NNT would have been the same if all the patients or only those classified as high risk had received thromboprophylaxis (NNT = 16 for both groups).

For the ONKOTEV score, the time-dependent Brier score was 0.14 (95% CI, 0.12–0.15), indicating well-calibrated predictions, while the time-dependent c-index was 0.53 (95% CI, 0.48–0.58), showing poor discrimination. Using a positivity threshold of 2 points, the 6-month cumulative incidence of VTE was 19.0% (95% CI, 14.4–24.2) in high-risk patients and 15.0% (95% CI, 12.0–18.4) in intermediate-risk patients (SHR, 1.05; 95% CI, 0.76–1.44; *p* = ns). The NTT would have been 16 if all the patients had received thromboprophylaxis versus 14 if only high-risk patients (ONKOTEV score ≥ 2) had been treated.

The items of the Khorana score and the PROTECHT score were not found to be significantly associated with the risk of VTE. The only covariate significantly associated with the risk of VTE was metastatic disease (SHR 1.56; 95% CI, 1.17–2.07; *p* = 0.002) from the ONKOTEV score (Table 4).

## 4. Discussion

In this large BACAP cohort of newly diagnosed PC patients receiving outpatient chemotherapy, the discriminatory performance of the Khorana, the PROTECHT, and the ONKOTEV scores to predict VTE at 6 months was poor, with c-indexes ranging from 0.50 to 0.53. When used dichotomously, none of these scores identified a subset of PC patients at significantly higher risk of VTE. These results do not support the use of any of these RAMs to guide thromboprophylaxis decisions in this specific patient population, who would derive the greatest clinical benefit from refined VTE risk prediction [27].

Our findings regarding the poor performance of the Khorana score in PC patients are consistent with previous studies [6,9,15,28,29,30,31,32]. In line with a recent large retrospective cohort of PC patients undergoing chemotherapy [9], we found a similar 6-month cumulative incidence of VTE in the Khorana intermediate-risk (16.5%) and high-risk (16.1%) categories. Half of the VTE events occurred in the Khorana intermediate-risk group (65.8% of patients), highlighting the low sensitivity of this RAM in PC patients at that stage of diagnosis and treatment. Even though the PROTECHT [13] and the ONKOTEV [14] RAMs incorporate additional relevant items, along with all previous Khorana score items, such constructs did not improve the VTE risk stratification in our study population. None of these scores have been derived or validated to predict VTE in a single-site cancer population, which may partly explain their poor performance in PC patients.

Three independent cohort studies, including only 2–11% of PC patients [13,33,34], evaluated the PROTECHT score in various cancer populations with locally advanced or metastatic disease. The PROTECHT score showed poor discriminatory performance in all studies, with c-indexes ranging from 0.59 to 0.61 [13,33,34]. In our study, the PROTECHT score showed limited applicability in newly diagnosed PC patients receiving chemotherapy, since 99.0% of them were classified as high risk for VTE.

The ONKOTEV score was externally validated in a retrospective single-center cohort of 165 PC patients [15], suggesting that this RAM might improve VTE risk stratification in PC patients. At a median follow-up of 6.3 months, the cumulative incidence of VTE was less than 10% in patients with an ONKOTEV score < 2, 41.8% in those with an ONKOTEV score of 2, and 70.6% in patients with an ONKOTEV score > 2. The c-index of the model was not reported. Other limitations were noticeable: patients with VTE events at enrollment were not excluded (39.2% of VTE events), only 66.1% of patients received chemotherapy at any time during follow-up, and 24.2% underwent surgical resection. The competing risk of death was not accounted for, leading to an overestimation of the risk of VTE. In contrast, the ONKOTEV score performed poorly in our cohort of PC patients (c-index of 0.53), where only patients with newly diagnosed PC who received outpatient chemotherapy were included if they did not have VTE at enrollment. As found when using the Khorana score, half of the VTE events occurred in the ONKOTEV intermediate-risk group (65.9% of patients), and the 6-month cumulative incidence of VTE was similar in the ONKOTEV intermediate-risk (15%) and high-risk (19%) categories.

The reported incidence of VTE in PC patients varies from 5% to 57% [4,5,6,7,8,9,10,28,29,30,35,36,37,38,39,40,41,42], depending on the study design, population, and follow-up duration. Our study provides a contemporary estimate of the 6-month risk of VTE in the new diagnosis of PC patients of any stage when receiving neoadjuvant chemotherapy. The 6-month cumulative incidence of VTE was 3-fold higher than that reported in recent registry-based cohort studies [3,32], and higher than the cumulative incidence of death during this period. Most VTE events occurred within 6 months after PC diagnosis, a critical period that warrants to consider the adequate use of primary thromboprophylaxis [6,8,9]. Recently, an estimated risk of VTE > 8–10% was proposed as a threshold for primary thromboprophylaxis in outpatients with cancer undergoing chemotherapy so that they may derive the best net clinical benefit [43]. In the present BACAP study cohort, regardless of the score used, the cumulative incidence of VTE at 6 months was greater than 10%, even in patients considered at intermediate risk for VTE. These results confirm that all PC patients scheduled to receive neoadjuvant chemotherapy should be offered primary thromboprophylaxis in the absence of contraindication to anticoagulant therapy.

An updated meta-analysis of the benefit of thromboprophylaxis in patients with locally advanced or metastatic PC undergoing chemotherapy reported a ~69% relative reduction in the risk of symptomatic VTE, without an increase in the risk of major bleeding [11]. Accordingly, the ITAC [44] and ESMO [45] clinical practice guidelines explicitly recommend thromboprophylaxis in all ambulatory patients with locally advanced or metastatic PC undergoing chemotherapy in the absence of contraindications or of a high risk of bleeding. The implementation of these guidelines into clinical practice is urgently needed to translate the evidence-based benefits from thromboprophylaxis.

This study is the first prospective cohort study to compare the performance of several RAMs for VTE in a large representative sample of newly diagnosed PC patients receiving chemotherapy with a relatively high number of VTE events. We did not evaluate the Khorana, PROTECHT, and ONKOTEV scores beyond 6 months because their performance was shown to decline over time [33,34]. All VTE events were adjudicated by trained adjudicators. We performed competing risk analyses to limit overestimation due to the competing risk of death. In addition, the inclusion of participants from 15 different centers with homogeneous clinical practices increases the generalizability of our findings. Although the Khorana, the PROTECHT, and the ONKOTEV scores were not available for 18% of the enrolled patients, multiple imputation was used to minimize bias due to missing data. Some limitations should be considered. We did not evaluate the COMPASS-CAT score because it was specifically developed for patients with breast, colorectal, lung, and ovarian cancer [46]. We did not evaluate other RAMs such as the CATS score [47], the CATS/MICA score [48], and the ONCOTHROMB score [49], nor the RAM proposed by Li et al. [50], as these models either incorporate biomarkers or genetic variants that were not routinely available in our patient population or were published after completing our study. More than half of the VTE events were incidentally diagnosed, while the Khorana and the PROTECHT scores were primarily derived from observed symptomatic VTE events. Nonetheless, both symptomatic and incidental VTE events are associated with worse prognosis in PC patients [4,5,6,9,29], and these RAMs have similar performance in predicting symptomatic or incidental VTE events [51].

## 5. Conclusions

This first prospective head-to-head comparison of the Khorana, the PROTECHT, and the ONKOTEV scores in PC patients receiving ambulatory chemotherapy showed similar poor performance of these RAMs in predicting the risk for VTE at 6 months. We suggest a more pragmatic approach, based on cancer stage (i.e., advanced stage), as recommended by the most recent ITAC [44] and ESMO [45] clinical practice guidelines, since the efficacy and safety of primary thromboprophylaxis has been clearly established in this subgroup of patients. There is considerable potential to improve risk prediction in PC patients, and future RAMs need to be more convincing.

## Figures and Tables

**Table 1:** Baseline characteristics of the 762 patients included in the study.

Characteristics	*n* = 762
Median age (IQR)	69 (60–76)
Male, *n* (%)	412 (54.1)
BMI	-
Median (IQR), kg/m^2^	23.5 (21.0–26.2)
≥35 kg/m^2^, *n* (%)	14 (1.9)
Missing, *n*	22
Performance status, *n* (%)	-
ECOG < 2	583 (87.3)
ECOG ≥ 2	85 (12.7)
Missing	94
Comorbidities, *n* (%)	-
Active smokers	383 (50.5)
Hypertension	301 (39.5)
Hyperlipidemia	168 (22.0)
Diabetes	193 (25.3)
Cardiac failure	15 (2.0)
Respiratory failure	12 (1.6)
History of VTE, *n* (%)	41 (5.4)
Primary tumor location, *n* (%)	-
Head	402 (53.5)
Isthmus	45 (6.0)
Body	100 (13.3)
Tail	77 (10.2)
Multiple	128 (17.0)
Missing	10
Stage, *n* (%)	-
Resectable tumor	46 (6.1)
Potentially resectable tumor	76 (10.0)
Locally advanced tumor	350 (46.2)
Metastatic tumor	286 (37.6)
Missing	5
Macroscopic vascular or lymphatic compression, *n* (%)	-
Yes	317 (47.3)
Missing	92
Scheduled chemotherapy within 6 months, *n* (%)	
Platinum-based therapy	465(61.0)
Gemcitabine-based therapy	258 (33.9)
Platinum- and gemcitabine-based therapy	29 (3.8)
Other chemotherapy	10 (1.3)
Hemoglobin, g/dL	
Median (IQR)	13.0 (11.9–14.0)
<10 g/dL, *n* (%)	32 (4.4)
Missing, *n*	38
Leukocyte count, ×10^9^/L	
Median (IQR)	7.5 (6.1–9.3)
>11 × 10^9^/L, *n* (%)	85 (11.7)
Missing, n	36
Platelet count, ×10^9^/L	
Median (IQR)	259 (205–318)
≥350 × 10^9^/L, *n* (%)	124 (16.3)
Missing, *n*	36
Median CA 19.9 (IQR), µmol/L	330.1 (46.7–2366.0)

Abbreviations: BMI, body mass index; ECOG, Eastern Cooperative Oncology Group; IQR, interquartile range; VTE, venous thromboembolism.

**Table 2:** Venous thromboembolic events within 6 months of pancreatic cancer diagnosis.

Events	Total Study Cohort (*n* = 762)
Total number of events, *n* (%)	73 (9.6)
Type of events, *n* (%)	-
Pulmonary embolism	17 (23.3)
Deep vein thrombosis	17 (23.3)
Visceral vein thrombosis	29 (31.5)
Catheter-related thrombosis	4 (5.5)
Combined venous thromboembolism events	6 (8.2)
Clinical presentation, *n* (%)	-
Symptomatic	32 (43.8)
Incidental	41 (56.2)

**Table 3:** Accuracy and discriminatory performance of the Khorana, PROTECHT, and ONKOTEV scores for predicting venous thromboembolism at 6 months.

	Khorana Score	PROTECHT Score	ONKOTEV Score
Brier score (95% CI)	0.14 (0.12–0.15)	0.14 (0.12–0.15)	0.14 (0.12–0.15)
Time-dependent c-index (95% CI)	0.50 (0.46–0.55)	0.50 (0.49–0.51)	0.53 (0.48–0.58)
Cumulative incidence of VTE, % (95% CI)	-	-	-
High-risk group	16.1 (11.4–21.5)	16.5 (13.9–19.3)	19.0 (14.4–24.2)
Intermediate-risk group	16.5 (13.4–19.8)	Not estimable *	15.0 (12.0–18.4)
SHR high- vs. intermediate-risk group (95% CI)	1.06 (0.77–1.45)	1.87 (0.29–12.05)	1.05 (0.76–1.44)

Abbreviations: CI, confidence interval; c-index, concordance index; SHR, sub-distribution hazard ratio; VTE, venous thromboembolism.

**Table 4:** Multivariable Fine–Gray regression analyses for VTE at 6 months.

	Khorana, SHR (95% CI)	PROTECHT, SHR (95% CI)	ONKOTEV, SHR (95% CI)
Platelet count			
<350 × 10^9^/L	Ref	Ref	
≥350 × 10^9^/L	1.07 (0.73–1.55)	1.08 (0.74–1.58)	
Hemoglobin level	-	-	-
≥10 g/dL	Ref	Ref	-
<10 g/dL	1.35 (0.72–2.52)	1.35 (0.72–2.55)	-
Leukocyte count	-	-	-
≤11 × 10^9^/L	Ref	Ref	-
>11 × 10^9^/L	0.99 (0.62–1.57)	0.98 (0.61–1.56)	
Body mass index	-	-	-
<35 kg/m^2^	Ref	Ref	
≥35 kg/m^2^	1.43 (0.58–3.56)	1.44 (0.57–3.59)	
Gemcitabine therapy	-	-	
No	-	Ref	-
Yes	-	1.26 (0.70–2.24)	
Platinum-based therapy	-	-	-
No	-	Ref	
Yes	-	1.18 (0.65–2.13)	
Khorana score	-	-	-
≤2	-	-	Ref
>2	-	-	0.98 (0.72–1.35)
Previous VTE	-	-	-
No	-	-	Ref
Yes	-	-	1.37 (0.79–2.37)
Metastatic disease	-	-	-
No	-	-	Ref
Yes	-	-	1.56 (1.17–2.07)
Macroscopic vascular compression	-	-	-
No	-	-	Ref
Yes	-	-	0.79 (0.59–1.06)

Abbreviations: CI, confidence interval; Ref, reference group; SHR, sub-distribution hazard ratio; VTE, venous thromboembolism.

## Data Availability

The original contributions presented in this study are included in the article/Supplementary Material. Further inquiries can be directed to the corresponding author(s).

## Supplementary Materials

The following supporting information can be downloaded at: https://www.mdpi.com/article/10.3390/cancers17040597/s1; Table S1: Risk assessment models for venous thromboembolism in pancreatic cancer patients; Table S2: Venous thromboembolic events during a median follow-up of 40 months; Table S3: Univariate and multivariate cox proportional hazards regression analysis of risk factors for venous thromboembolism in the study population; Table S4: Distribution of the Khorana, PROTECHT, and ONKOTEV scores in the study population; Figure S1: Cumulative incidence of venous thromboembolism and death from pancreatic cancer diagnosis; Figure S2: Cumulative incidence of venous thromboembolism in high- and intermediate-risk groups by the (A) Khorana score at the threshold of 3 and the (B) ONKOTEV score at the threshold of 2.

## Appendix A. Members of the BACAP Consortium

Barbara Bournet, Cindy Canivet, Louis Buscail, Nicolas Carrère, Fabrice Muscari, Bertrand Suc, Rosine Guimbaud, Corinne Couteau, Marion Deslandres, Pascale Rivera, Anne-Pascale Laurenty, Nadim Fares, Karl Barange, Janick Selves, Anne Gomez-Brouchet, The CHU and the University of Toulouse, Toulouse, France; Bertrand Napoléon, Bertrand Pujol, Fabien Fumex, Jérôme Desrame, Christine Lefort, Vincent Lepilliez, Rodica Gincul, Pascal Artru, Léa Clavel, Anne-Isabelle Lemaistre, Jean Mermoz Hospital, Lyon, France; Laurent Palazzo, Trocadéro Clinic, Paris, France; Jérôme Cros, The Department of Pathology, Beaujon Hospital and Paris 7 University, Clichy, France; Sarah Tubiana, The Biobank, Bichat Hospital and Université de Paris, Paris, France; Nicolas Flori, Pierre Senesse, Pierre-Emmanuel Colombo, Emmanuelle Samail-Scalzi, Fabienne Portales, Sophie Gourgou, Claire Honfo Ga, Carine Plassot, Julien Fraisse, Frédéric Bibeau, Marc Ychou, The Cancer Institute and the University of Montpellier, Montpellier, France; Pierre Guibert, Christelle de la Fouchardière, Matthieu Sarabi, Patrice Peyrat, Séverine Tabone-Eglinger, Caroline Renard, The Léon Bérard Cancer Center, Lyon, France; Guillaume Piessen, Stéphanie Truant, Alain Saudemont, Guillaume Millet, Florence Renaud, Emmanuelle Leteurtre, Patrick Gele, The Department of Digestive Surgery, the CHU and the University of Lille, Lille, France; Eric Assenat, Jean-Michel Fabre, François-Régis Souche, Marie Dupuy, Anne-Marie Gorce-Dupuy, Jeanne Ramos, The CHU and the University of Montpellier, Montpellier, France; Jean-François Seitz, Jean Hardwigsen, Emmanuelle Norguet-Monnereau, Philippe Grandval, Muriel Duluc, Dominique Figarella-Branger, La Timone Hospital and the University of Marseille, Marseille, France; Véronique Vendrely, Clément Subtil, Eric Terrebonne, Jean-Frédéric Blanc, Etienne Buscail, Jean-Philippe Merlio, The CHU and the University of Bordeaux, Bordeaux, France; Dominique Farge, Jean-Marc Gornet, Daniela Geromin, Saint Louis Hospital and Université de Paris, Paris, France; Geoffroy Vanbiervliet, Anne-Claire Frin, Delphine Ouvrier, Marie-Christine Saint-Paul, Philippe Berthelémy, Chelbabi Fouad, The CHU and University of Nice, nice, France; Stéphane Garcia, Nathalie Lesavre, Mohamed Gasmi, Marc Barthet, The CHU Nord Hospital and the University of Marseille, Marseille, France; Vanessa Cottet, INSERM UMR866 and the University of Dijon, Dijon, France; Cyrille Delpierre, INSERM UMR1027 and the University of Toulouse, Toulouse France.
