# Supplementary material for: Risk Assessment Models for Predicting Venous Thromboembolism in Patients with Pancreatic Cancer
Source: Cancers (Basel). 2025 Feb 10;17(4):597. doi: 10.3390/cancers17040597 (PMC11853169; doi:10.3390/cancers17040597)

Supplementary material

Table S1. Risk assessment models for venous thromboembolism in pancreatic cancer patients.

	**Khorana score**	**PROTECHT score**	**ONKOTEV score**
Pancreatic cancer	+2	+2	-
Prechemotherapy platelet count ≥ 350 x10^9/L	+1	+1	-
Prechemotherapy hemoglobin level <10 g/dL and/or use of ESAs	+1	+1	-
Prechemotherapy leukocyte count >11 x10^9/L	+1	+1	-
Body mass index ≥35 kg/m^2	+1	+1	-
Gemcitabine-based therapy	-	+1	-
Platinum-based therapy	-	+1	-
Khorana score>2	-	-	+1
Previous VTE	-	-	+1
Metastatic disease	-	-	+1
Macroscopic Vascular compression	-	-	+1

Abbreviations: BMI, body mass index; ESA, erythropoiesis stimulating agents; VTE, venous thromboembolism.

Table S2. Venous thromboembolic events during a median follow-up of 40 months (95% CI, 34.3-46.8).

	**Total study cohort (n=762)**
Total number of events, n (%)	195 (25.6)
Type of events, n (%)	-
Pulmonary embolism	41 (21.0)
Deep vein thrombosis	52 (26.7)
Visceral vein thrombosis	67 (34.4)
Catheter-related thrombosis	19 (9.7)
Combined VTE events	16 (8.2)
Clinical presentation, n (%)	-
Symptomatic	86 (44.1)
Incidental	109 (55.9)

Abbreviations: n, number; VTE, venous thromboembolism

Table S3. Univariate and multivariate cox proportional hazards regression analysis of risk factors for venous thromboembolism in the study population.

		Univariate analysis		Multivariate analysis		
	HR	95% CI	*P-value*	HR	95% CI	*P-value*
Age (≥70 years *versus* <70 years)	**0.65**	**0.49-0.88**	**0.004**	**0.63**	**0.47-0.84**	**0.002**
Sex (female *versus* male)	0.87	0.66-1.16	0.345			
Primary Tumor location			0.233			
Isthmus (*versus* head)	1.30	0.75-2.25				
Body (*versus* head)	1.09	0.71-1.69				
Tail (*versus* head)	1.64	1.04-2.59				
Overlapping lesion (*versus* head)						
Stage			**<0.001**			
Locally advanced (*versus* resectable)	**2.26**	**0.83- 6.16**				
Metastatic (*versus* resectable)	**4.16**	**1.53-11.32**		**2.11**	**1.22-3.63**	**0.007**
Diabetes (yes *versus* no)	**0.73**	**0.52-1.03**	**0.076**			
Hypertension (yes *versus* no)	0.90	0.67-1.20	0.482			
Hyperlipidemia (yes *versus* no)	1.13	0.82-1.55	0.467			
Alcohol consumption (yes *versus* no)	1.06	0.76- 1.48	0.729			
Tobacco consumption (yes *versus* no)	0.85	0.571.28	0.176			
Previous VTE (yes *versus* no)	**1.91**	**1.13- 3.25**	**0.016**			
Obesity (BMI > 30 kg/m^2) (yes *versus* no)	1.01	0.61-1.68	0.593			
Recent surgery (<3 months) (yes *versus* no)	2.18	1.07-4.43	0.031			
Bedridden patient (yes *versus* no)	1.41	0.66-3.01	0.369			
Lower limbs varicose (yes *versus* no)	0.99	0.64-1.55	0.974			
Previous thoracic central venous catheter (yes *versus* no)	**1.89**	**0.97-3.71**	**0.062**			
Cardiac failure (yes *versus* no)	1.53	0.68-3.44	0.309			
Macroscopic vascular compression at diagnosis (yes *versus* no)	0.79	0.58-1.06	0.119			
CA 19.9 (≥37 *versus* <37 IU/mL)	1.02	0.68-1.54	0.919			
Hemoglobin (<13.5 *versus* ≥13.5 g/dL)	0.91	0.68-1.22	0.534			
Leucocytes (<11 *versus* ≥11 $x10^9$/)	1.16	0.74-1.83	0.515			
Platelets (>450 *versus* ≤ 150 $x10^9$/l)	0.66	0.26-1.68	0.426			
ALAT (≤72 *versus* >72 IU/L)	0.98	0.71-1.35	0.906			
ASAT (≤30 *versus* >30 IU/L)	1.04	0.77-1.42	0.789			
γ-GT (≤64 *versus* >64 IU/L)	1.24	0.9-1.72	0.194			
Bilirubin (≤22 *versus* > 22µmol/L)	0.90	0.65-1.24	0.502			

**Alkaline Phosphatase (≤126 *versus* >126 IU/L)**	0.94	0.69-1.28	0.783			
**First line chemotherapy**			0.190			
**Gemcitabine (*versus* Platinum-based)**	1.30	0.97-1.76				
**Gemcitabine + Platinum-based (*versus* Platinum-based)**	1.02	0.50-2.10				
**Other (*versus* Platinum-based)**	0.36	0.05-2.62				

Results were derived with Cox's proportional hazards models with a time-dependent covariate. Variables associated with a *P-value* <.10 in univariate analysis were introduced in the multivariate model. Then, backward and stepwise selection strategies of the variables were performed. The selection procedure consists of removing variables having the largest *P-value* greater than .10 from the model. Some variables that were statistically significant in the univariate analysis were no more significant in multivariate analysis. The final multivariate model presented corresponds to the final results of the multivariate model. Hazard ratio are reported only for variables with significant effects in the multivariate model.

Abbreviations: ALAT, alanine aminotransferase; ASAT, aspartate aminotransferase; BMI, body mass index; CA 19.9, carbohydrate antigen 19-9; γ-GT, gamma-glutamyl transferase HR, Hazard Ratio; 95% CI, 95% confidence interval; VTE, venous thromboembolism.

Table S4. Distribution of the Khorana, PROTECHT and ONKOTEV scores in the study population.

Score	n (%)
**Khorana score**	**-**
2 points	501 (71.9)
3 points	154 (22.1)
4 points	36 (5.2)
5 points	6 (9.3)
Missing	65
Positivity threshold 3 points	
Intermediate risk of VTE (2 points)	501 (65.8)
High risk of VTE (≥3 points)	196 (25.7)
**PROTECHT score**	**-**
2 points	7 (1.0)
3 points	476 (68.3)
4 points	165 (23.7)
5 points	42 (6.0)
6 points	7 (1.0)
Missing	65
Positivity threshold 3 points	-
Intermediate risk of VTE (2 points)	7 (1.0)
High risk of VTE (≥3 points)	690 (99.0)
**ONKOTEV score**	-
0 point	131 (21.2)
1 point	276 (44.7)
2 points	172 (27.8)
3 points	37 (6.0)
4 points	2 (0.3)
Missing	144
Positivity threshold 2 points	-
Intermediate risk of VTE (<2 points)	407 (65.9)
High risk of VTE (≥ 2 points)	211 (34.1)

Abbreviations: n, number; VTE, venous thromboembolism.

Figure S1. Cumulative incidence of venous thromboembolism and death from pancreatic cancer diagnosis.

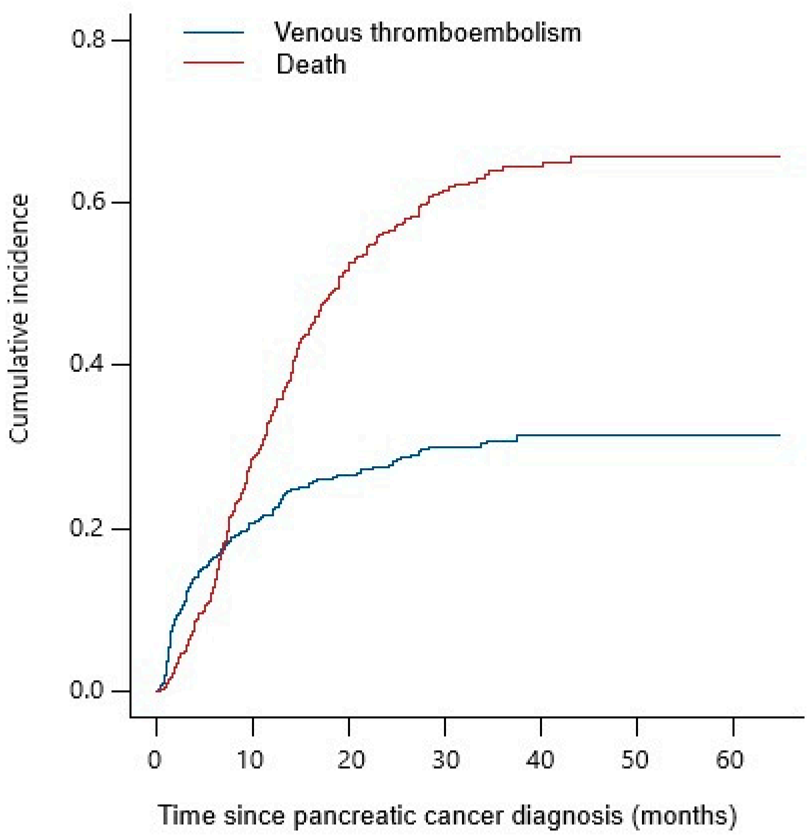

Figure S2. Cumulative incidence of venous thromboembolism (VTE) in high-and intermediate-risk groups by the (A) Khorana score at the threshold of 3, (B) ONKOTEV score at the threshold of 2. The cumulative incidence of VTE could not be estimated for the intermediate-risk group of the PROTECHT score (7 patients). Abbreviations: SHR, subdistribution hazard ratio; VTE, venous thromboembolism.

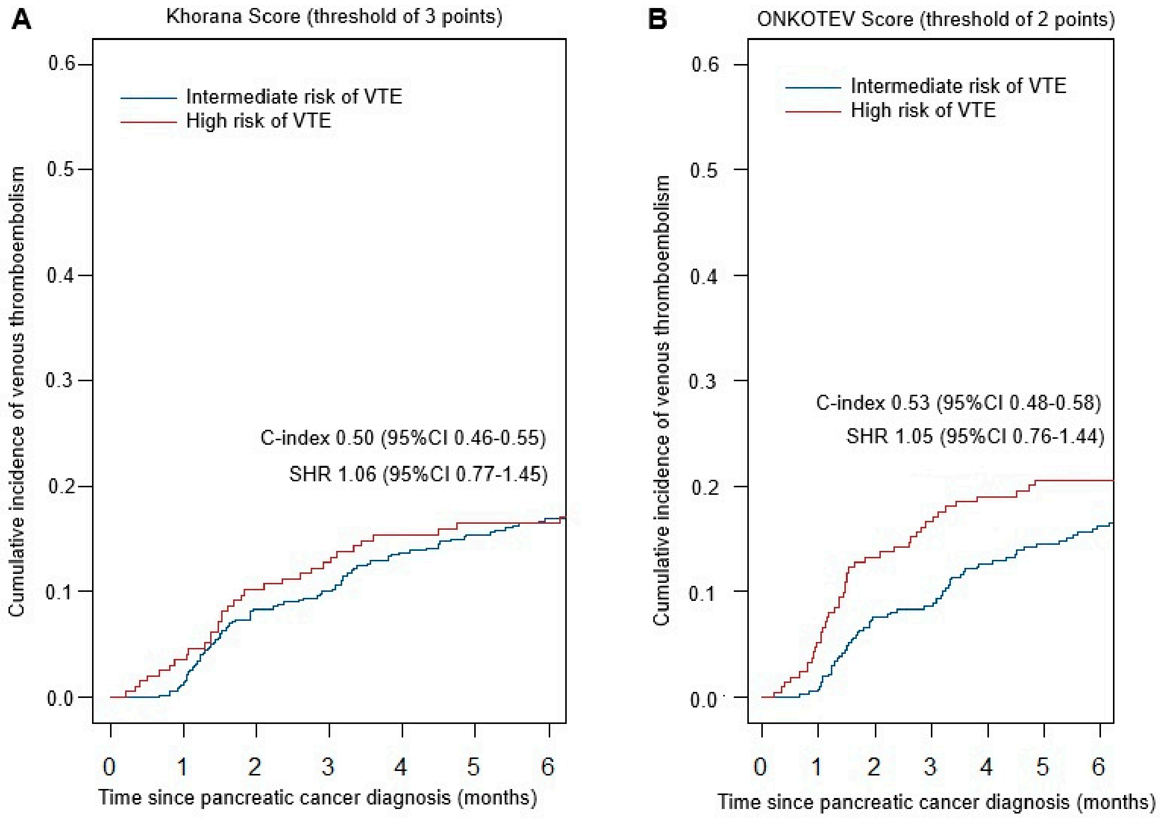

Supplement: Supplementary file 1 [file cancers-17-00597-s001.zip › cancers-3405817-supplementary.pdf]
